# Imaging of Vulva Syringoma With Reflectance Confocal Microscopy

**DOI:** 10.3389/fmed.2021.649438

**Published:** 2021-02-24

**Authors:** Lin Feng, Yan Lin, Leilei Wang, Hongxiao Chen, Min Gao, Huaxu Liu, Hongyu Yang

**Affiliations:** ^1^Chongqing Hospital of Traditional Chinese Medicine, Chongqing, China; ^2^Shandong Provincial Institute of Dermatology and Venereology, Shandong First Medical University & Shandong Academy of Medical Sciences, Jinan, China; ^3^Shandong Cancer Hospital and Institute, Shandong First Medical University & Shandong Academy of Medical Sciences, Jinan, China; ^4^Linyi People's Hospital, Linyi, China; ^5^Department of Pathology, St. Vincent Evansville Medical Center, Evansville, IN, United States

**Keywords:** *in vivo* imaging, reflectance confocal microcopy, vulva, syringoma, histology, differential diaenosis

## Abstract

**Objectives:** To investigate the application of reflectance confocal microscopy (RCM) imaging in diagnosis of vulva syringoma.

**Methods:** Patients with lesions suspicious of syringoma on vulva were enrolled in the study. After informed consent was taken, the lesions were photographed and imaged with RCM. The features of the lesion in confocal images were then analyzed and compared with the biopsy findings for histology correlation.

**Results:** Eleven cases in total were included in the study. The typical RCM features observed in syringoma are the presence of round to oval high refractive, and relatively monomorphous mass of varying sizes in the superficial and middle dermis, usually surrounded with 1–2 layers of light-dark line structures, which were further confirmed by histological evaluation. Ten cases showed classic features of syringoma and 1 case exhibited milia in RCM images.

**Conclusions:** Syringoma has distinct features in RCM imaging, which correlates well with histological findings, highlighting the potential role of RCM in the diagnosis and differential diagnosis of vulva syringoma.

## Introduction

Syringoma is a benign sweat gland tumor derived from eccrine ducts and it occurs predominantly in women at puberty or later in life (1). The typical lesions of syringoma present as multiple, small, firm, skin-colored papules, usually 1–3 mm in diameter, and symmetrically distributed on the periorbital region (1, 2). Although the most common site of localized involvement is periorbital, syringoma in other areas have also been reported, including the vulva, penis, palms, scalp, and axillae (2, 3). The classification criterion of syringoma proposed by Friedman and Butler (4) was based on clinical features and consists of 4 variants: localized, familial (5), a form associated with Down Syndrome (DS), and a generalized variant, including multiple and eruptive syringoma (6).

The characteristics of lesions on the eyelids and forehead can be easily identified, while lesions in other areas especially on the vulva were not easily identifiable and the definitive diagnosis was usually made by histological examination. However, the invasive biopsy procedure for histology analysis reduced the compliance of the patients with suspicious vulva lesions, therefore developing a non-invasive skin imaging modality in the diagnosis and differential diagnosis of syringoma seems more appealing to potential patients.

The reflectance confocal microscopy (RCM) is a non-invasive skin imaging modality with high “cellular” resolution (7), which could compare the cellular changes of epidermis and superficial dermis *in vivo* in real time, and its accuracy is comparable with the histological evaluation of syringoma. In this study, we investigated the role of RCM in the diagnosis of vulva syringoma.

## Patients and Methods

**Figure 1:**
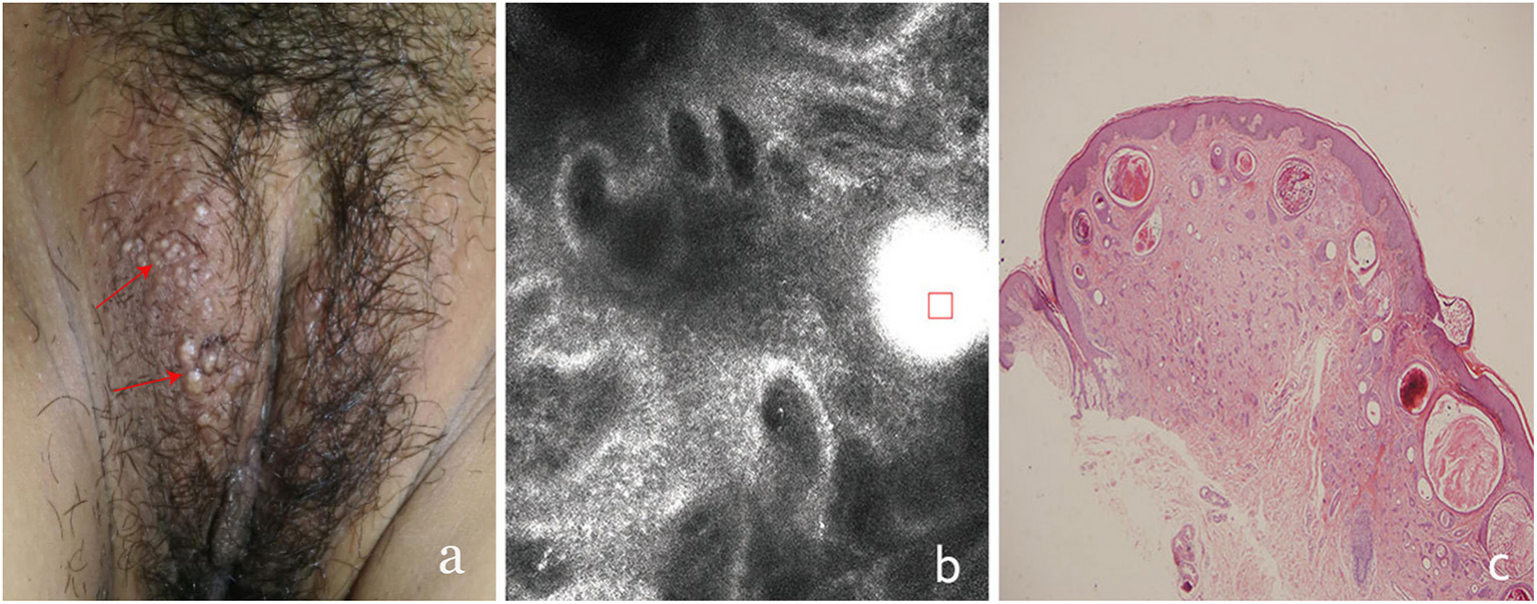
The clinical presentation, RCM findings and histology of vulva syringoma (case 1). **(a)**, the clinical image of case 1, **(b)**, the RCM image findings; **(c)**, the histology findings. The presence of round to oval high refractive, and relatively monomorphous mass of varing sizes (the red square)was prensent in the superficial dermis, with 1–2 layers of surrounding light-dark line structure (red arrowhead) in **(b)**.

**Figure 2:**
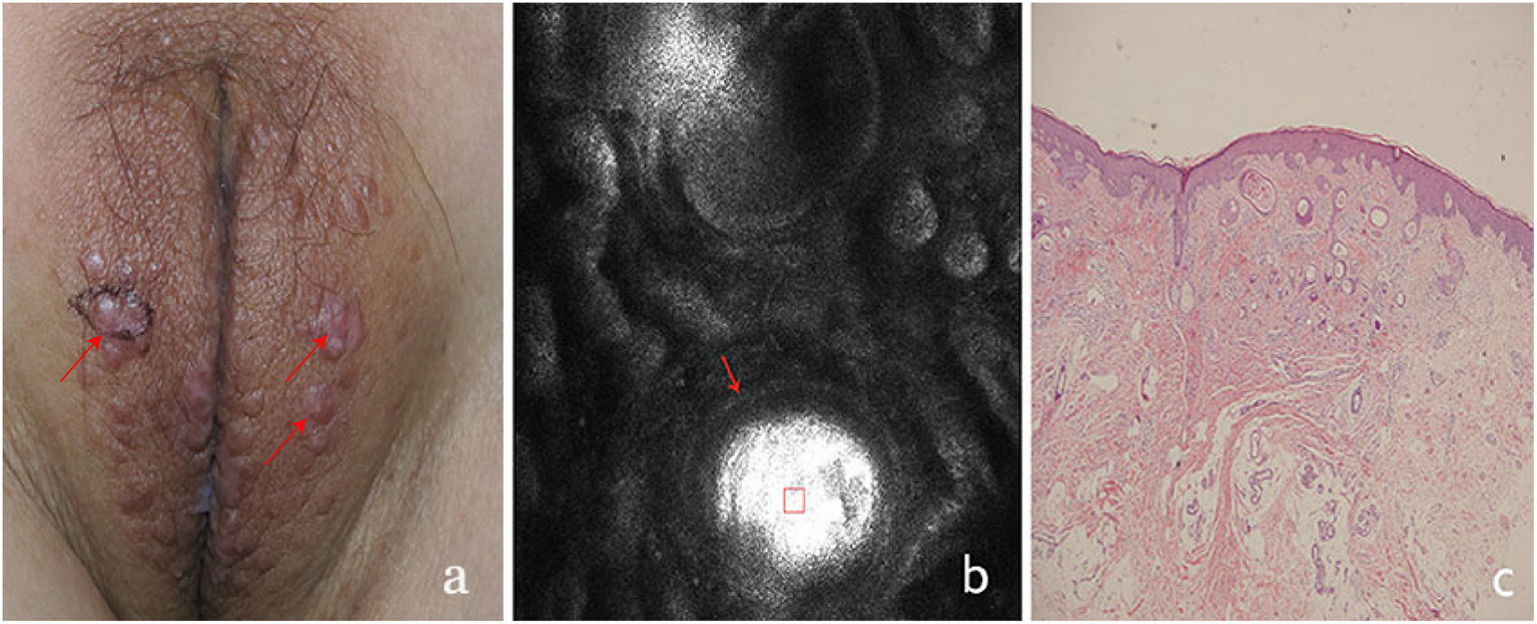
The clinical presentation, RCM findings and histology of vulva syringoma (case 2). **(a)**, the clinical image of case 2, **(b)**, the RCM image findings; **(c)**, the histological findings. The presence of round to oval high refractive, and relatively monomorphous mass of varing sizes (the red square) was present in the superficial dermis, with 1–2 layers of surrounding light-dark line structure (red arrowhead) in **(b)**.

**Figure 3:**
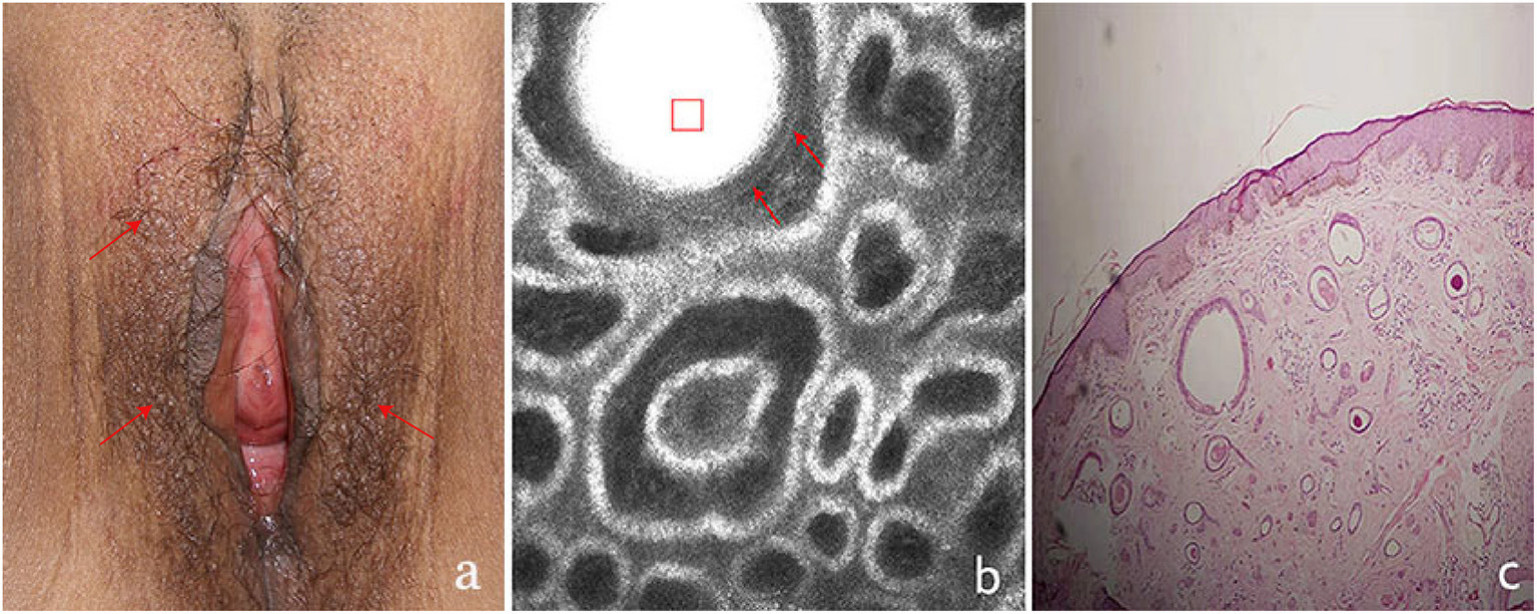
The clinical presentation, RCM findings and histology of vulva syringoma (case 3). **(a)**, the clinical image of case 3, **(b)**, the RCM image findings; **(c)**, the histological findings. The presence of round to oval high refractive, and relatively monomorphous mass of varing sizes (the red square)was present in the upper dermis, with 1–2 layers of surrounding light-dark line structures (red arrowhead)in **(b)**.

**Figure 4:**
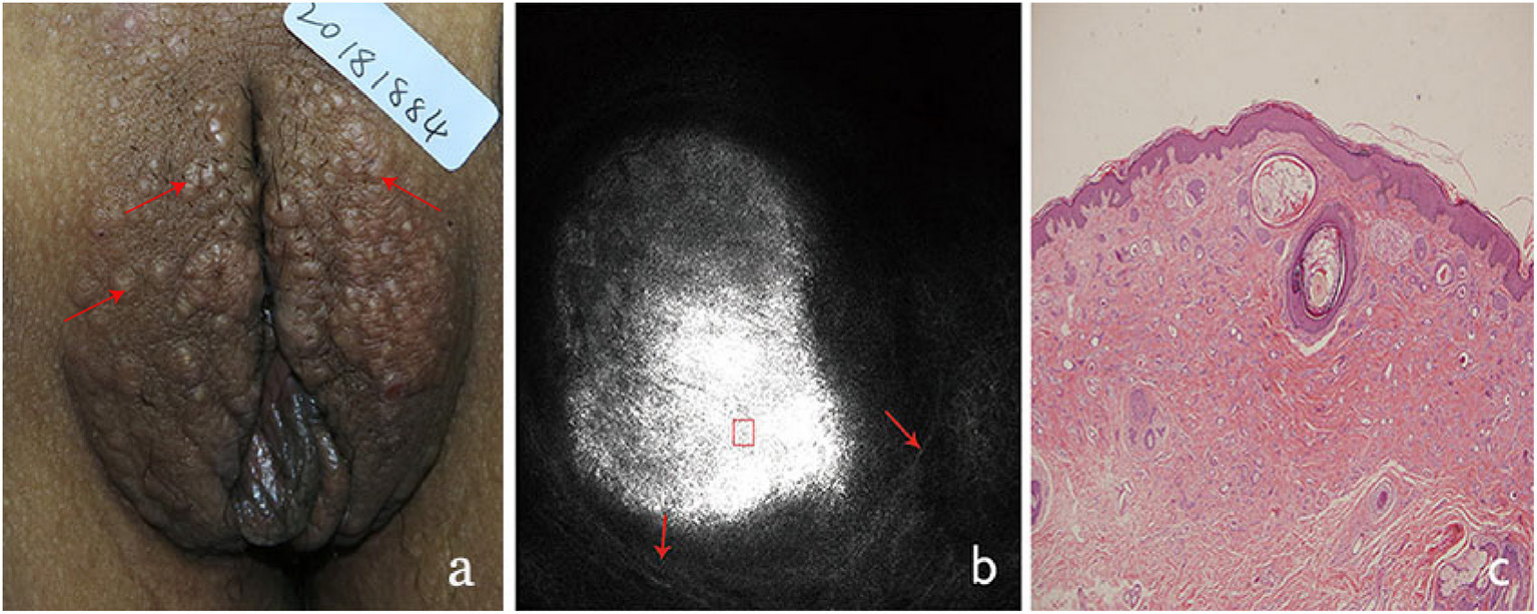
The clinical presentation, RCM findings and histology of vulva syringoma (case 4). **(a)**, the clinical image of case 4, **(b)**, the RCM image findings; **(c)**, the histological findings.

The study had been approved by Ethics Committee of Shandong Cancer Hospital and Institute and was conducted from 2018 to 2019. Cases with lesions suspicious of syringoma on vulva (Figures 1a, 2a, 3a, 4a) were enrolled in the study. After written informed consent was signed, the lesions were photographed and imaged by a commercially available, reflectance mode confocal microscope (Vivascope 1500; Caliber Imaging & Diagnostics, Inc. formerly Lucid, Inc., Rochester, NY, USA). The captured horizontal images in a 500 × 500 μm field, and viva-block image of 3 × 3 mm at different layers were obtained. A detailed description of the technique and the device has been published previously (7, 8). After imaging with RCM, the same lesions were biopsied and fixed in phosphate-buffered neutral formalin, embedded in paraffin, and stained with hematoxylin-eosin (HE), then analyzed using an optical microscope to investigate the accuracy of RCM imaging.

## Results

A total of 11 female adults were enrolled in the study (Table 1). The average age of the cases was 30.5 years old, and the average duration of the lesion was 17 months.

**Table 1:** The age and medical history of the patients enrolled in the study.

**Case number**	**Age**	**Medical history (month)**	**RCM imaging (+ or –)**	**Histology results (+ or –)**
1	23	12	+	+
2	32	16	+	+
3	44	20	+	+
4	21	30	+	+
5	32	23	+	+
5	34	14	+	+
7	25	11	+	+
8	27	17	+	+
9	28	9	+	+
10	31	13	+	+
11	39	22	-(milia)	-(milia)
Average	30.5	17		

*+ with significant changes of syringoma in RCM or histology images*.

*− other changes*.

On clinical examination, multiple small, light brown papules are scattered in the vulva area (Figures 1a, 2a, 3a, 4a), or in a “beaded” distribution along the outer margin of labia Majora. The main characteristics of lesions from the patients observed by RCM were very similar. There were no significant cellular changes in superficial epidermis, and the keratinocytes appeared regular and small, and the architectural pattern of the honeycomb was similar with the peri-lesional normal skin. Deeper level imaging showed brighter keratinocytes in the basal cell layer in lesional zone and the melanin contents in the basal cell layer were also increased compared with the adjacent normal skin.

The most characteristic changes were observed in the upper and middle dermis. Evenly distributed varying sizes of round to oval structures show high refractive mass with surrounding light-dark lines (Figures 1b, 2b, 3b, 4b). In comparison, there was just mild to moderate refracted collagen in upper and middle dermis in adjacent non-lesional areas.

**Figure 5:**
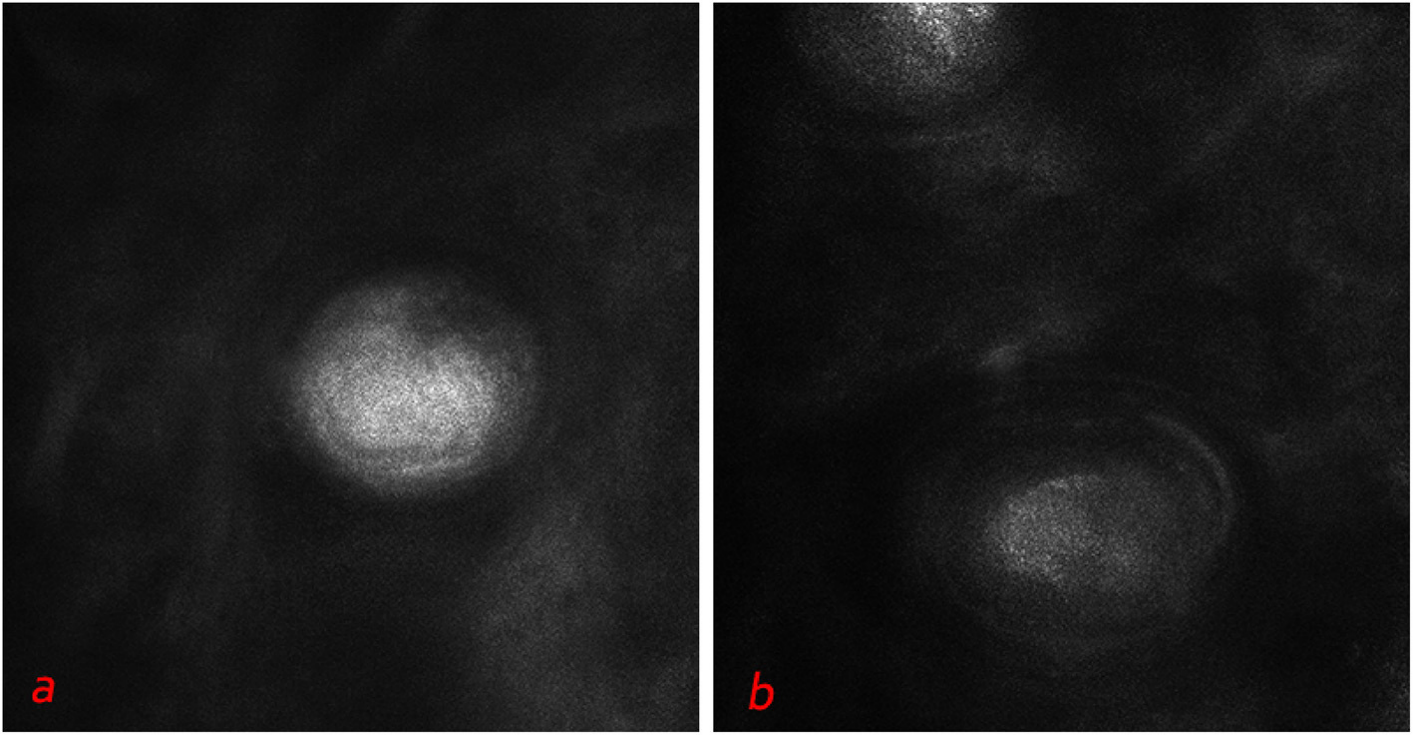
Deeper level imaging of syringoma of vulva area **(a,b)** showed that the refraction of the round structures tends to be slightly decreased as they go deep.

The definitive histological diagnoses were all confirmed by biopsy finding in all cases studied, which show classic findings of syringoma with superficial to mid-dermal nests of eccrine ducts and tadpole-like structures embedded in fibrous stroma. clear cell changes of epithelial cells were occasional present. The ductal lumina are filled with an amorphous, periodic acid-Schiff-positive material (Figures 1c, 2c, 3c, 4c), Deeper level imaging showed that the refraction of the round structures tends to be slightly decreased as they go deep (Figures 5a,b).

## Discussion

Syringoma occurs more frequently in adult female with distinct histopathologic features (1, 2). In cases with characteristic clinical presentation, syringoma can be easily diagnosed clinically. For lesions on unusual sites such as the genital area, the clinical manifestation was usually atypical. They need to be confirmed by biopsy and histological diagnosis. However, the invasive biopsy procedure would reduce the compliance of the patients, and a non-invasive method for the diagnosis and differential diagnosis of syringoma on vulva sites is definitely sought after by various medical professionals.

The application of dermoscopy imaging in diagnosis of syringoma has been reported and dermoscopy may contribute to the imaging diagnosis of syringoma by revealing multiple bright cystic enlargements in dermis (9). While comparing with dermoscopy, RCM provides more details of those lesions studied with more sensitivity and specificity.

The RCM is also non-invasive and dynamically shows the cellular-level morphology in human skin *in vivo* (7, 10). Imaging is based on the detection of single back scattered photons from the optical section and contrast due to the relative variations in refractive indices and sizes of organelles and micro-structures (7). The cellular changes of the lesion in epidermis and superficial dermis could be imaged and compared with that of the adjacent normal skin. The RCM had been widely used in the screening of skin tumors, such as melanoma (10, 11), basal cell carcinoma (12), and actinic keratosis (13). In recent years, the RCM was reported to be useful in the diagnosis, differential diagnosis, and follow-up of pigmented and inflammatory skin disorders (14, 15) with high sensitivity and specificity.

The typical changes of syringoma are in mostly confined to superficial and mid-dermis, which is in the imaging depth of RCM. And we found the presence of round to oval, high refractile, and relatively evenly distributed substances of varying sizes in the superficial and mid-dermis with surrounding light-dark line structures based on RCM imaging to be characteristic for synringomas, which were confirmed by histology results. The well-demarcated high refractile substances noted in confocal image correlate with the amorphous, periodic acid-Schiff-positive material in the ductal lumina in histological findings, while the surrounding 1–2 layers of light-dark line structures seen in confocal images correlate with the lining epithelial cells and myoepithelial cells seen in histological images.

Vulva areas can be affected by many papular skin lesions, which should be differentiated from syringoma, those entities include milia, Fox-Fordyce disease, epidermal cysts, lichen planus, and lichen simplex chronicus (9, 16, 17). The confocal images of milia showed relatively high refractile, but unevenly distributed keratin in superficial dermis (18), which is more superficial compared with syringoma in confocal image findings. The major difference between syringoma and milia in confocal images is the “evenly” refractile substance in dermis and the presence of surrounding 1–2 layers of light and dark lines noted in the former and not present in the latter (18). Our observations are all confirmed with the histological findings. As to other cutaneous adnexal tumors, the RCM could also detect the changes around the follicles and cellular changes in epidermis and superfical dermis, which is useful in the screening and differential diagnosis of those lesions.

A recent case report of syringoma considered the dark areas surrounded by rims of epithelia as the characteristic of syringoma based on RCM findings (19). They interpreted the encircling hollow dark areas as lumina. However, in our study, we identified the round to oval, high refractile, and relatively monomorphous substances of varying sizes in the superficial and middle dermis to represent lumina. And future studies with a larger sample size coupled with histological analysis is needed to confirm our findings.

Our preliminary study showed the novel features of syringoma in RCM imaging, which could be useful as a non-invasive “*in vivo*” tool in the screening and differential diagnosis of suspicious vulva syringomatous lesions. The appropriate clinical application of RCM or other skin imaging methods will potentially reduce the invasive biopsy procedure and related medical cost, and in the end benefit the patients.

## Data Availability Statement

The raw data supporting the conclusions of this article will be made available by the authors, without undue reservation.

## Ethics Statement

The studies involving human participants were reviewed and approved by The ethic committe of Shandong Provincial Hospital for Skin Diseases. The patients/participants provided their written informed consent to participate in this study.

## Author Contributions

LF, YL, and LW collected the cases and che clnical and RCM data. HC collected the histology data. MG and HL designed the study and prepared the manuscript. HY revised the manuscript. All authors contributed to the article and approved the submitted version.

## Conflict of Interest

The authors declare that the research was conducted in the absence of any commercial or financial relationships that could be construed as a potential conflict of interest.
